# Effects of Persistent Introgression on Mitochondrial DNA Genetic Structure and Diversity in the *Apis cerana cerana* Population

**DOI:** 10.3390/insects17010128

**Published:** 2026-01-22

**Authors:** Shujing Zhou, Miao Jia, Yidan Long, Bingfeng Zhou, Yinan Wang, Zhining Zhang, Yue Wang, Danyang Zhang, Xinjian Xu, Xiangjie Zhu

**Affiliations:** 1 College of Bee Science, Fujian Agriculture and Forestry University, Fuzhou 350002, China; sjzhou118@fafu.edu.cn (S.Z.); 12306044001@fafu.edu.cn (M.J.); 15095868700@163.com (Y.L.); 000q060008@fafu.edu.cn (B.Z.); 15770520248@163.com (Y.W.); zhangzhining@fafu.edu.cn (Z.Z.); 12407044012@fafu.edu.cn (Y.W.); 18769918668@163.com (D.Z.); 2 Honeybee Research Institute, Fujian Agriculture and Forestry University, Fuzhou 350002, China

**Keywords:** *Apis cerana cerana*, introgression, population structure, genetic diversity, mtDNA, WGS

## Abstract

Continuous human-mediated introduction of colonies and queens promotes genetic introgression and reshapes the genetic diversity and structure of local honeybee populations. According to reports, multiple non-native honeybee colonies and queens have been introduced into the DL region, leading to continuous genetic introgression. Here, we assessed the effects of continuous introgression on indigenous *Apis cerana* in the DL region using mtDNA and genome-wide SNP markers. We sequenced the mitochondrial tRNA ^leu^-COII from 217 individuals sampled at 7 DL sites and identified 26 haplotypes defined by 18 polymorphic sites. The ΦST values indicated no internal differentiation within the *Apis cerana* populations in the DL region. Phylogenetic, network, ABBA-BABA test, and *f*3 statistic suggested introgression from both northern and southern sources. The *f*4-ratio indicates that approximately 16% of the ancestry in the DL group is derived from the Aba group. Genetic diversity varied widely within the DL region (*Hd*: 0.2907–0.8220; *π*: 0.0009–0.0038; *K*: 0.3140–1.3980), indicating different stages of introgression. The genetic structure within the DL group appears to be unstable, necessitating long-term monitoring of evolutionary processes and genetic diversity dynamics in *A. c. cerana* for further insights.

## Simple Summary

The prevalence of non-native subspecies introductions in the Daloushan (DL) region raises questions about their specific impact on population structure and population diversity. This study examined the tRNA ^leu^-COII sequence of 217 samples in the region, identified genetic lineage admixture, and detected two distinct types of introgression within *Apis cerana cerana* from southern and northern origins. This finding was confirmed through gene flow analysis using whole-genome data. Furthermore, there is inconsistency in the internal genetic structure of the DL population, with varying levels of genetic diversity. This suggests that continuous gene flow has kept the *Apis cerana* populations in the region in a state of instability, emphasizing the necessity of early protection for honeybees with local genetic characteristics.

## 1. Introduction

With the advancement of economic development, increased commercial activities, and improved transportation conditions, the interregional transportation of honeybee colonies and queens is expanding. This is leading to heightened genetic exchange between different subspecies or geographic populations [1]. Additionally, the natural mating behavior of bees complicates genetic management. Mature drones, whether from artificially reared colonies or wild populations, gather in specific aerial areas during mating flights to mate with virgin queens [2]. Due to this biological trait, alien genetic material can swiftly infiltrate the genetic pool of native bee colonies, leading to introgression.

Research indicates that gene penetration can impact the genetic composition of native populations. Studies have shown changes in the genetic makeup of bee populations in Sicily, Italy, and the Southern Ural region in Russia due to the introduction of non-native subspecies [3,4]. When genes from external sources are not well-suited to the local ecological environment, they can endanger the genetic stability and long-term adaptability of indigenous populations. The local subspecies *Apis mellifera mellifera* in Germany has faced near-local extinction due to the extensive introduction of commercially bred queens [5,6,7]. Given the inherent challenge of completely removing external individuals, the genes they harbor may persist within a population in a neutral or recessive fashion, thereby influencing genetic homogeneity and local subspecies characteristics.

Mitochondrial haplotype and genome-wide assessments of gene flow are key components in current genetic introgression studies. In the field of genetic introgression detection, mitochondrial DNA (mtDNA), characterized by maternal inheritance, lack of recombination, and high mutation rate, is widely utilized in studies of population phylogenetics, population genetic structure, and gene introgression [8,9,10,11]. The mitochondrial tRNA ^leu^-COII intergenic region is a unique non-coding region specific to the honeybee. Significant genetic structural differences are observed among different distribution regions of *Apis mellifera*, indicating rich genetic diversity that can effectively distinguish between different geographic populations of *Apis mellifera* [12,13,14]. The length of the sequence from 41–97 bp makes it cost-effective for sequencing and provides a substantial amount of foundational genetic data. Therefore, this fragment serves as an effective molecular marker for studying gene introgression, genetic structure, and genetic diversity within bee species [15,16,17,18]. Mitochondrial haplotype network construction and phylogenetic analyses are commonly used approaches to investigate maternal lineage differentiation and mitochondrial introgression [19,20]. Furthermore, there are numerous gene flow detection methods based on genome-wide data. The ABBA-BABA statistic (D statistic) and related statistics such as the *f*4-ratio are commonly used to assess evidence of gene flow between populations or closely related species [21]. The admixture *f*3 (C; A, B) statistic tests whether population C is a mixture of populations A and B, with significantly negative values (Z scores < −3) regarded as strong evidence of admixture [22].

The Daloushan (DL) region is a favorable habitat with abundant nectar sources, serving as a significant native range for *Apis cerana cerana*, which thrives in substantial numbers in the wild [23]. In recent years, due to poverty alleviation and rural revitalization policies, there has been a rapid expansion in beekeeping scale in Guizhou Province. Multiple non-native honeybee colonies and queens were introduced. From 2007 to 2019, the number of *Apis cerana* colonies in the province increased from around 60,000 to 510,000 [24,25,26,27]. According to reports, the introduction of honeybee colonies and queens has resulted in a decrease in the local bee population, reduced resistance, diminished foraging abilities, and increased aggressiveness [23,27]. This has transformed the region into an ideal “natural experimental model” for studying the impact of sustained gene flow on local populations. We aim to jointly investigate the impact of gene introgression on the DL region using mitochondrial sequences and whole-genome data.

## 2. Materials and Methods

### 2.1. Sampling and DNA Extraction

**Figure 1:**
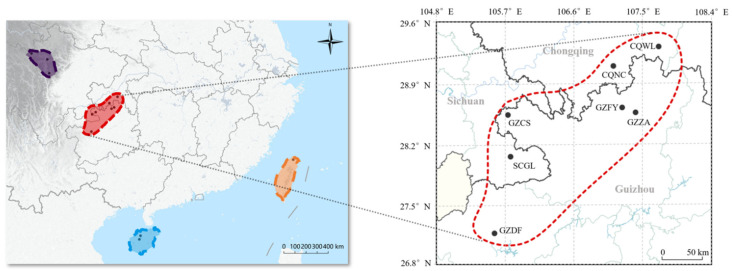
Information on sampling sites. The black markers in red circle represent the seven sampling points in the DL region: CQWL, Wulong; CQNC, Nanchuan; GZFY, Fuyan; GZZA, Zheng’an; GZDF, Dafang; GZCS, Chishui; SCGL, Gulin. The purple (*A. c. abansis*), blue (*A. c. hainana*), and orange (*A. c. taiwanensis*) areas are hypothesized to be the source populations for introgression.

A total of 217 individuals, from 7 sampling sites (31 individuals for each site), were sampled in the DL region (Figure 1; Table S1). In addition to the above samples, this study also collected three populations, namely *A. c. abansis* (Aba group), *A. c. hainana* (HAN group), and *A. c. taiwanensis* (TW group), each consisting of 31 individuals, as potential outgroup sources. Each individual represents a single colony. Samples were taken from inside the hives, placed in absolute ethanol, and then stored at −20 °C until DNA extraction. Genomic DNA was extracted from the thorax of 217 individuals using the EZup column animal genomic DNA purification kit (Sangon Biotech, Shanghai, China).

### 2.2. tRNA ^leu^-COII Intergenic Region

PCR analysis of the tRNA ^leu^-COII intergenic haplotypes was performed using primers E (5′-TCAGGGTATTCATAGGATCA-3′) and H (5′-TTTAATATGGCAGAATAGTG-3′) in a final volume of 50 μL: 19 μL of sterile ddH_2_O, 2 μL of each primer (10 pmol/μL), 25 μL of PrimeSTAR Max, and 2 μL of DNA template. The primers were designed by Primer Premier 5 [28] and were synthesized by Fuzhou Shangya Biological Co., Fuzhou, China. The PCR conditions were as follows: initial denaturation at 95 °C for 5 min, followed by 30 cycles of denaturation at 95 °C for 30 s, annealing at 50 °C for 30 s, and elongation at 72 °C for 1 min, with a final elongation at 72 °C for 8 min. The PCR products were sent to Shangya Biological Co., Ltd. (Fuzhou, China) for direct sequencing in both directions using the Sanger method.

### 2.3. Analysis of mtDNA Data

The tRNA ^leu^-COII sequences were aligned manually using Clustal X 1.83 [29], and all mitochondrial sequences were blasted on NCBI. Summary statistics, including polymorphic sites, number of haplotypes (*H*), haplotype diversity (*Hd*), nucleotide diversity (*π*), the average number of nucleotide differences (*K*), and the number of polymorphic sites, were calculated for each of the seven populations using DnaSP 5.10 [30].

In order to examine the sources of introgression for *A. c. cerana*, we conducted analyses on a larger dataset including the Aba group, HAN group and TW group. This allowed us to obtain a more complete overview of the genetic structure of *A. c. cerana* in the DL region. Genetic differentiation among the DL sampling sites and the reference subspecies was estimated using *Fst* values, calculated by Arlequin 3.5.2.2 [31]. The ΦST values and analysis of molecular variance (AMOVA) were calculated using 20,000 permutations.

Relationships among haplotypes and their relative proportions in DL were inferred using the median-joining network algorithm [32,33], as implemented in NETWORK 10.0 (Fluxus Engineering, Clare, UK). To examine the phylogenetic relationships among haplotypes based on the tRNA ^leu^-COII region [19,20], we constructed a maximum-likelihood (ML) tree in IQ-TREE 3.0.1 [34], using the sequence (AP018149) from Malaysia as an outgroup [35].

### 2.4. Genomic Analysis

To obtain more genetic information, we selected 10 samples from each of the Aba, HAN, and DL groups, for a total of 30 populations for genome analysis. All DNA samples were constructed as genomic DNA libraries and high-throughput sequenced by Novogene (Beijing, China) using an Illumina PE150 system (Illumina, San Diego, CA, USA). Adapters were removed and low-quality sequencing fragments were filtered out by conducting data quality control with fastp 0.23.4 [36]. All clean reads were mapped to the reference genome of *Apis cerana* (GCF_029169275.1) using BWA 0.7.19 [37] with the parameter: “mem -t 4 -k 32 -M”. PCR or optical duplicates were removed using SAMtools 0.1.19 [38] with the command: “rmdup”. Single-nucleotide polymorphisms (SNPs) were called using Genome Analysis Toolkit (GATK) 4.6.20 [39] and filtered with a depth between 4 and 1000 (4 < DP < 1000), mapping quality (MQ) >20.0, minor allele frequency (MAF) >0.01, and a maximum missing genotype rate <0.05 to obtain high-quality SNPs.

LD for each population was assessed by calculating the squared allele frequency correlation (R^2^) statistic using the PopLDdecay package 3.43 [40]. The maximum distance between two SNPs was set to 5 kb. The Perl script Plot_MultiPop.pl in PopLDdecay was used to calculate and visualize the LD decay.

Population structure was analyzed using ADMIXTURE 1.3.0 [41] to estimate individual ancestry based on SNP genotypes through maximum likelihood. Principal component analysis (PCA) was conducted using pLink2 1.9.0-b.8 [42], and the top two major components were plotted using the R package ggplot2 4.0.1 [43].

To further confirm genetic introgression, ABBA-BABA analysis (Patterson’s D) and *f*4-ratio were performed using Dsuite 0.5 [21]. Patterson’s D measures the excess of shared derived alleles between P3 and either P1 or P2 based on a tree model of four groups as (((P1, P2), P3, O), where (P1, P2), P3 is DL and O is the outgroup. The D statistic is 0 when there is no gene introgression between P1, P2, and P3, and will be positive or negative if there is gene introgression between P2 and P3 or between P1 and P3. The *f*4-ratio provides a quantitative estimate of the extent of introgression from the source population into the target population. Additionally, we calculated the *f*3 statistic using ADMIXTOOLS 8.0.2 [22] to further confirm gene flow among the DL, Aba, and HAN groups.

## 3. Results

### 3.1. Genetic Variation in the DL

The sequencing and alignment of the tRNA ^leu^-COII region in *A. c. cerana* from the DL, a fragment of around 352 bp was obtained. The average content of bases A, T, C, and G was found to be 40.95%, 44.58%, 9.84%, and 5.00%, respectively. The average A + T content was 85.53%, and the average C + G content was 14.84%, indicating a pronounced AT-skew. A total of 18 polymorphic sites were detected, including 13 parsimony-informative sites and 5 singleton mutation sites.

**Figure 2:**
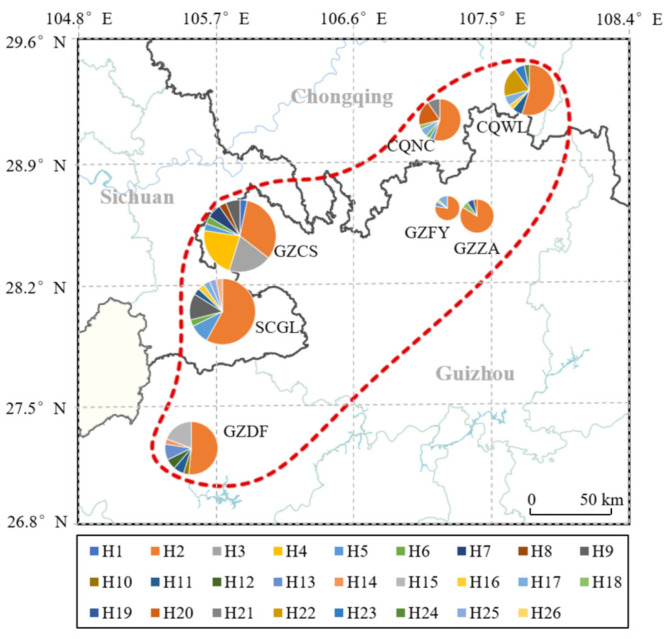
The haplotype distribution of the DL. A total of 26 haplotypes (H1–H26) that were detected in DL. Each color represents a different haplotype, with the size of the circle indicating the frequency of each haplotype. Larger circles correspond to higher frequencies.

A total of 26 haplotypes (Accession ID: Table S5) were identified and labeled as H1-H26 (Figure 2). The most common haplotype was H2 (Acmt01001), which was present in all *A. c. cerana* populations and was the most frequent (58.99%) haplotype in all sampling sites (Table S2). Haplotypes H2, H5, H6, H9, H11, H16, H17, H18, and H20 were distributed in more than two sites, accounting for 78.80% of the total samples. Specifically, haplotypes H5 (GZCS, GZFY, CQNC, SCGL) and H17 (GZFY, CQNC, CQWL, SCGL) were found in four sites, while haplotypes H6 (GZCS, CQNC, SCGL), H11 (GZDF, CQWL, SCGL), and H17 (GZFY, CQWL, SCGL) were found in three sites. Haplotypes H9 (GZCS, SCGL), H18 (GZZA, CQNC), and H20 (GZZA, CQNC) were identified in two sites each.

In the DL region, a total of 12 unique haplotypes were found, representing 21.20% of the collected samples. These haplotypes are distributed among 5 sampling sites: GZCS (5 unique haplotypes: H1, H3, H4, H7, H8), GZDF (5 haplotypes: H10, H12, H13, H14, H15), CQWL (3 haplotypes: H22, H23, H24), SCGL (2 haplotypes: H24, H25), GZZA (1 haplotype: H19), and CQNC (1 haplotype: H21). Notably, GZFY did not exhibit any unique haplotypes.

### 3.2. Genetic Differentiation

The AMOVA results (Table 1) showed that 95.13% of the genetic variation was attributable to differences between the DL group and the three outgroups, indicating extremely strong among-group differentiation (ΦCT = 0.9513, *p* < 0.001) and supporting the reliability of our outgroup selection. Overall, the genetic differentiation was also very high (ΦST = 0.9564, *p* < 0.001). In contrast, differentiation among populations within the DL group was low, suggesting no pronounced genetic subdivision within the DL populations (ΦSC = 0.1047, *p* < 0.001).

**Figure 3:**
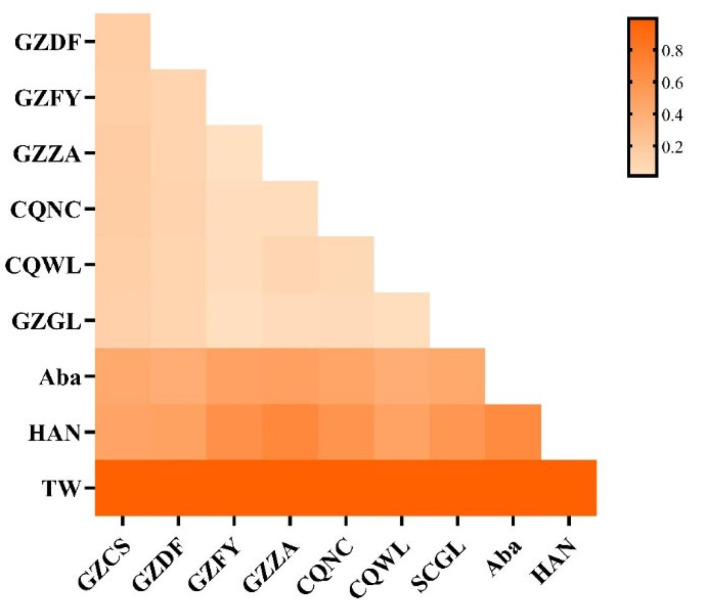
Pairwise *Fst* matrix of honey bee populations in the DL region. The heatmap shows the matrix of pairwise *Fst* values among DL sampling sites (e.g., GZDF, GZFY, GZZA, CQNC, CQWL, GZGL) and the hypothesized donor populations (Aba, HAN, TW). Color intensity reflects the magnitude of *Fst*.

Analysis of inter-population *Fst* (Figure 3; Table S3) revealed significant genetic differentiation between *A. c. cerana* and external populations, with *Fst* values ranging from 0.3987 to 0.9924. Within the DL geographical populations, there was no apparent genetic differentiation, although GZCS and GZDF showed slightly higher *Fst* values compared to other sampling sites. The highest genetic differentiation was observed between GZCS and other sites, with *Fst* values ranging from 0.1300 to 0.1604, followed by GZDF with *Fst* values of 0.0941 to 0.1075. The *Fst* values between SCGL, GZFY, GZZA, CQNC, and CQWL ranged from 0.0140 to 0.0855, indicating low levels of differentiation.

### 3.3. Network Analysis

**Figure 4:**
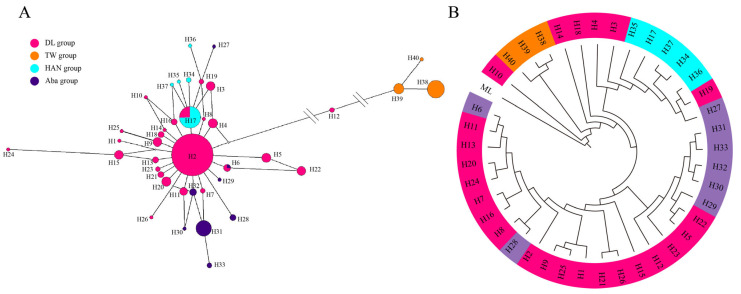
Haplotype relationships of *A. c. cerana* in the DL region and reference populations based on mtDNA tRNA ^leu^-COII. (**A**): Median-joining network of haplotypes. Different colors represent distinct groups, with circle size indicating haplotype frequency, where larger circles correspond to higher frequencies. (**B**): Genetic relationships among DL sampling sites and reference subspecies using the Maximum Likelihood (ML). Model selection was performed using ModelFinder (implemented in IQ-TREE 3.0.1) under the best-fit substitution model HKY + F+G4. Different colors indicate different populations, and the sequence from Malaysia as an outgroup.

The haplotype frequencies and relationships of *A. c. cerana* in the DL region were clarified using the median-joining network (Figure 4A). Four main clusters were identified, representing the DL group, TW group, HAN group, and Aba group. Haplotype H2, centrally positioned among the DL haplotypes, displayed the highest connectivity with other haplotypes and was found in all sampling locations, indicating its role as the ancestral haplotype in the DL region. The shared haplotype H17 was positioned at the center of the HAN group, connecting with DL haplotypes H3 and H19. Haplotype H31 was situated at the center of the Aba group, along with haplotype H7 in this cluster. The DL group exhibited the greatest genetic divergence from the TW group, with no shared haplotypes between them.

### 3.4. Evolutionary Analysis Based on Haplotypes

The maximum likelihood phylogenetic tree, constructed based on the tRNA ^leu^-COII sequence, depicts the relationships among haplotypes (Figure 4B). ModelFinder selected the HKY + F + G4 model as the optimal substitution model for the ML tree construction. The resulting tree delineates major clades encompassing all haplotypes, with three reference populations forming distinct clusters, while haplotypes from the DL region exhibit a notably non-monophyletic distribution pattern. The haplotypes within the DL group exhibit a clustered pattern of multiple origins: some DL haplotypes (e.g., H3, H4, H17, H19) cluster with HAN group haplotypes, while other DL haplotypes cluster with Aba group haplotypes (e.g., H5, H6, H8, H11, H22). Apart from the aforementioned haplotypes clustering with the reference groups, the remaining DL haplotypes form a relatively independent clustered branch on the tree, indicating the preservation of regional maternal lineage units with distinctive characteristics in the DL region. Furthermore, some haplotypes within the DL population do not strictly cluster based on geographical origins but instead show a mixed distribution with different reference populations (HAN and Aba groups), reflecting the complexity of maternal genetic backgrounds in the DL region; this topological feature aligns overall with the phylogenetic relationships revealed by haplotype network analysis (Figure 4).

### 3.5. Genetic Diversity

**Figure 5:**
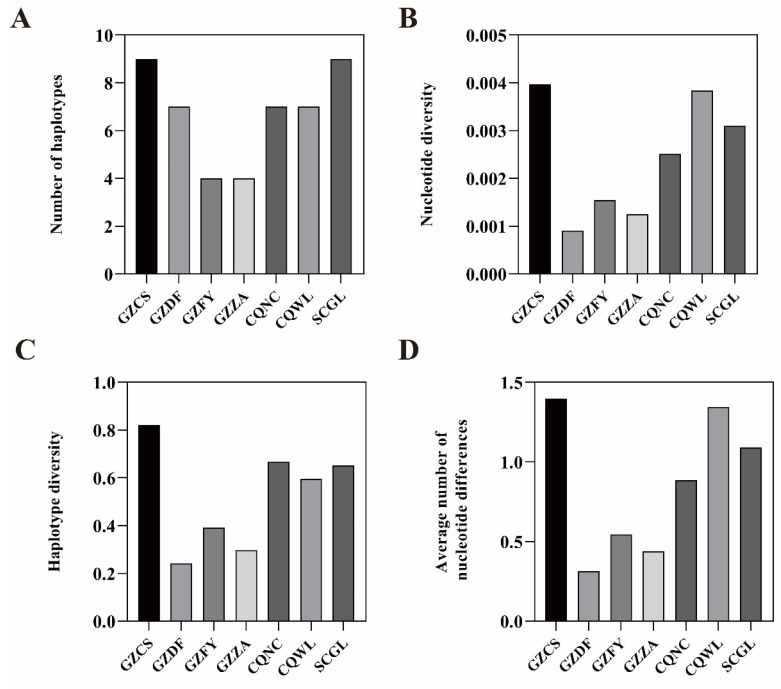
Comparison of genetic diversity among sampling sites. (**A**): Number of haplotypes (**B**): Nucleotide diversity (**C**): Haplotype diversity (**D**): Average number of nucleotide differences.

Genetic diversity estimates for each sampling site in the DL region are presented in Figure 5 (Table S4). Genetic diversity varied considerably across sites, with haplotype diversity (*Hd*) ranging from 0.2907 to 0.8220, nucleotide diversity (*π*) ranging from 0.0009 to 0.0038 and the average number of nucleotide differences ranging from 0.3140 to 1.398. The overall average haplotype diversity was 0.5240. Among the sites, GZCS (0.8220), CQNC (0.6667), SCGL (0.6520), and CQWL (0.5960) exceeded this average, while GZDF (0.2430) exhibited the lowest haplotype diversity.

Similarly, the overall average nucleotide diversity was 0.00245. Sites GZCS (0.0040), CQWL (0.0038), SCGL (0.0031), and CQNC (0.0025) showed higher than average nucleotide diversity, whereas GZDF had the lowest value (0.0009). GZFY and GZZA exhibited moderate levels of diversity.

### 3.6. Genomic Genetic Structure

**Figure 6:**
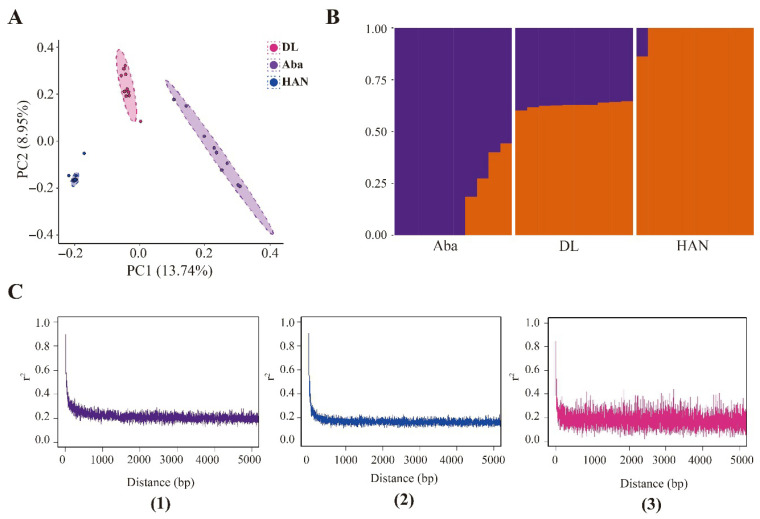
Population genetic diversity and variation of honey bees. (**A**) Principal component analysis (PCA) plot showing Aba (purple), DL (pink), and HAN (blue) samples on PC1 and PC2. (**B**) Population genetic structure of three groups (K = 2). Different colors indicate different groups. (**C**) LD differences among honey bee populations. LD decay is measured on the basis of the squared correlations of allele frequencies (R^2^) against the distance between polymorphic sites. (1) Aba group; (2) HAN group; (3) DL group.

PCA analysis using whole-genome SNP data demonstrated distinct genetic differentiation among the three populations (Figure 6A). The first principal component (PC1), which accounted for 13.74% of the total variance, effectively distinguished the HAN, DL, and AB populations. The second principal component (PC2), explaining 8.95% of the variance, showed considerable overlap between the AB and DL populations, indicating a higher degree of genetic similarity between these groups.

The results of the ADMIXTURE and LD decay analyses were consistent with the PCA results. The ADMIXTURE analysis (Figure 6B) revealed that DL individuals exhibit two main ancestral components, one shared with Aba and the other with HAN, indicating a clear admixture pattern. LD (r^2^) decayed rapidly with increasing physical distance in all three groups, reaching a low background level (Figure 6C). This suggests efficient historical recombination, a relatively large effective population size, and generally weak long-range LD. Interestingly, the DL group (Figure 6C-(3)) exhibited a slightly elevated and highly fluctuating LD plateau at intermediate to long distances, suggesting the presence of introgressed tracts of varying lengths throughout the genome.

### 3.7. Gene Introgression Test Between Subspecies

**Figure 7:**
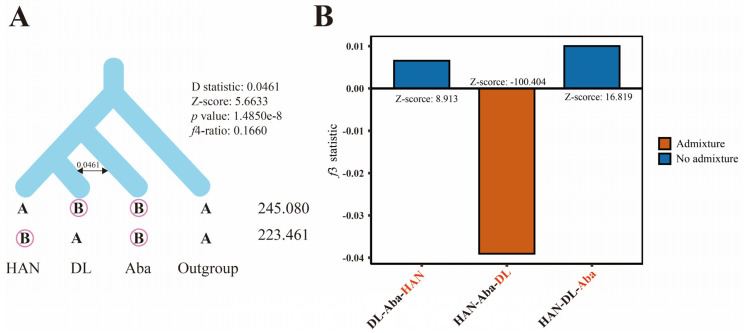
Gene flow test among the DL, HAN, and Aba groups. (**A**) ABBA-BABA analysis of the three groups, with the *f*4-ratio estimating the introgression proportion. The D statistic, Z-score, *p* value, and *f*4-ratio are shown. The numbers on the right indicate the counts of ABBA and BABA loci. (**B**) The *f*3 statistic tests for different population combinations. The bars show *f*3 statistics with Z-scores labeled. Orange indicates significantly negative *f*3 values consistent with admixture, whereas blue indicates no evidence of admixture.

To examine gene flow among the three *Apis* populations, we calculated the D-statistics using Dsuite (Figure 7A). The ABBA-BABA test, also referred to as the D-test (D-statistics), is a commonly used approach in population genetics for identifying significant deviations resulting from admixture events. The ABBA-BABA test was employed, with *A. dorsata* used as the outgroup. The analysis combination model is ([HAN, DL], Aba, Outgroup). Z-scores exceeding an absolute value of >3 indicate statistically significant gene flow. The results confirmed that gene flow was observed between DL samples and Aba samples. Additionally, the *f*4-ratio indicated that approximately 16.60% of the ancestry in the DL group derives from the Aba group.

To further confirm the gene flow between DL samples and the Aba and HAN groups, we calculated the admixture *f*3 statistic using Admixtools (Figure 7B). The admixture *f*3 analysis showed a significantly negative value (Z < −3) for the combination (HAN, Aba; DL), with *f*3= −0.0391 and Z = −100.4, indicating strong evidence that population DL represents a mixture of populations HAN (Type 1) and Aba (Type 2).

## 4. Discussion

This study demonstrates that, over the past decade, the *A. c. cerana* in the DL region has exhibited a typical genetic structure and genetic diversity characteristics shaped by the continuous introduction of colonies and queens. Mitochondrial DNA analysis revealed the presence of non-native haplotypes, while whole-genome resequencing further indicated the presence of hybridization signals at SNP loci, showing a significantly mixed pattern of genetic structure. Due to adaptive differences from introgressed genes, the different sampling sites in the DL group exhibit distinct levels of genetic diversity.

Both the maternal and nuclear datasets support widespread introgression in DL. At the mitochondrial level, the maximum-likelihood phylogeny and haplotype network link DL lineages to two major putative sources: a southern lineage represented by the HAN group (Type 1) and a northwestern lineage represented by the Aba group (Type 2). This multi-source scenario is corroborated by genome-wide SNP evidence, as the *f*4-ratio estimate indicates that approximately 16.60% of the ancestry in DL is derived from the Aba group. The concordance between mtDNA and SNP signals strengthens the inference that introgression in DL has been ongoing and geographically complex.

From an evolutionary perspective, whether alien alleles persist in the local gene pools depends largely on their fitness in the recipient environment. Introgression theory predicts that the removal rate of alien genes depends on their adaptability to the new environment [44,45,46,47]. When alien genes have low fitness, they are quickly eliminated [48]. Some introduced individuals may not survive in the local environment, leaving no offspring and resulting in undetectable alien genes in the local gene pool. In harsh conditions or prevalent diseases, the harmfulness of low-fitness alien genes increases [49]. These genes may require extended selection for elimination, leading to an initial increase in genetic diversity and eventually shaping a new genetic structure suited to the local environment. In the DL region, ongoing gene introgression prolongs the process of removing unfavorable alien genes, maintaining the local genetic structure in an unstable state.


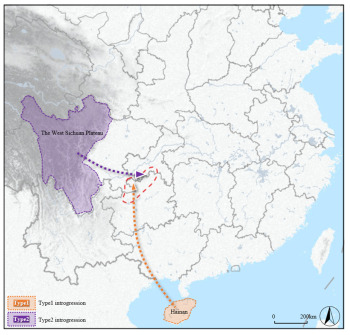


Our results are consistent with an ecological-compatibility explanation for the differential persistence of introgressed components. In the DL group, the haplotype shared with HAN (H17) accounted for 4.15% (9/217) of individuals, whereas the haplotype shared with Aba (H6) accounted for only 1.38% (3/217). Type 1 introgression likely originates from South and Southeast China, where a subtropical monsoon climate broadly resembles that of the DL region [50]; such similarity may facilitate retention and local spread of Type 1 components. By contrast, Type 2 introgression is plausibly associated with the western Sichuan Plateau [51], characterized by high elevation, low temperatures, and reduced precipitation, which differ markedly from the warm and humid conditions of DL. This climatic mismatch may reduce the fitness of Type 2 components in DL, increasing the probability of selective purging and limiting long-term persistence.

These patterns further imply that introgression can have stage-dependent effects on genetic diversity. While repeated introductions may elevate variation in the short term by adding novel haplotypes and alleles, subsequent selection against maladaptive components can lead to a decline after initial enrichment [52,53,54]. In addition, gene swamping may accelerate the erosion of native variation if hybrids or introgressed genomes are selectively removed, potentially eliminating linked indigenous alleles as collateral. The uneven distribution of diversity across DL populations therefore suggests that enrichment and erosion may operate simultaneously, with direct implications for the conservation of locally adapted lineages.

Accordingly, enhanced monitoring and management of colony and queen movements, along with conservation-oriented breeding and resource protection, will be essential to mitigate the potential impacts of continuous introgression on local genetic diversity and genetic integrity [55].

## 5. Conclusions

Continuous introgression plays a crucial role in shaping the genetic structure of *Apis cerana* in the DL region, possibly increasing short-term genetic variation while posing long-term risks of diluting or losing local genetic resources. Future work should quantify the time scale and intensity differences of introgression, assess the adaptive outcomes of various introgression types, and combine queen and colony management strategies to mitigate the potential effects of continuous introgression on local genetic diversity and integrity.

## Figures and Tables

**Table 1:** Analysis of Molecular Variance (AMOVA) for *A. cerana* Across Different Regions.

Source of Variation	d.f.	Sum of Squares	Variance Components	Percentage of Variation
Among groups	3	1732	11.59	95.13
Among populations within groups	6	15	0.06	0.51
Within populations	300	160	0.53	4.36
Total	309	1906	12.18	

Fixation Indices: ΦSC = 0.1047 (*p* < 0.001), ΦST = 0.9564 (*p* < 0.001), ΦCT = 0.9513 (*p* < 0.001).

## Data Availability

The original contributions presented in this study are included in the article. Further inquiries can be directed toward the corresponding authors.

## Supplementary Materials

The following supporting information can be downloaded at https://www.mdpi.com/article/10.3390/insects17010128/s1. Table S1: Information of sampling sites; Table S2: Frequency of Haplotypes; Table S3: *Fst* values; Table S4: Genetic diversity; Table S5: The accession IDs of haplotypes in this study.
